# Familial microdeletion 18p11.32 to 18p11.31 in a Chinese family with normal phenotype

**DOI:** 10.1186/s13039-022-00590-5

**Published:** 2022-03-24

**Authors:** Miaomiao Han, Lei Wei, Fang Liu, Xia Gao

**Affiliations:** 1 Department of Obstetrics and Gynecology, Maternal and Child Health Hospital of Dongxihu District, Wuhan, Hubei China; 2 Department of Center for Reproductive Medicine, TaiHe Hospital, Hubei University of Medicine, Shiyan, Hubei People’s Republic of China; 3 Department of Gynecology, Huangshi Love & Health Hospital Affiliated To Hubei Polytechnic University, Huangshi, Hubei People’s Republic of China; 4 Department of Obstetrics, Renmin Hospital, Hubei University of Medicine, Shiyan, Hubei People’s Republic of China

**Keywords:** CMA, Microdeletion 18p11.32 to 18p11.31, Prenatal diagnosis, Unbalanced chromosome abnormality (UBCA)

## Abstract

**Background:**

Chromosomal imbalances of several megabasepair in size are normally deleterious for the carrier. Still, rarely reported are so-called “unbalanced chromosome abnormalities” (UBCAs), which are either gains or losses or equally large genomic regions, but the affected person is not or only minimally clinically affected. The knowledge of such UBCAs is imperative also in chromosomal microarray analysis (CMA) or noninvasive prenatal testing (NIPT).

**Case presentation:**

A maternally inherited del(18)(p11.32p11.31) was identified in a over two generations in a Chinese family with normal phenotype. The affected region encompasses 19 genes, among which *TGIF1* is expressed in fetal and adult nervous system. *TGIF1* deletions and /or mutations have been seen in cases with holoprosencephaly but also non-affected individuals, suggesting incomplete penetrance and variable expressivity.

**Conclusions:**

Deletions in the terminal region of chromosome 18 short arm have been reported previously in clinically healthy persons. Here a further family with an UBCA in 18p11.3 is added to the literature, suggesting a careful genetic counselling in comparable, especially prenatal cases.

## Background

Besides whole chromosome gains or losses, microdeletions and microduplications are in the focus of prenatal diagnostics [1]. Nowadays especially noninvasive prenatal testing (NIPT) is more and more applied to exclude such chromosomal imbalances in the developing child [2].

Besides clearly disease causing chromosomal imbalances there are also rare cases of euchromatic variants [3] and also the unbalanced chromosome abnormalities (UBCAs) [4]. Euchromatic variants do not cause clinical symptoms and are often nothing else than cytogenetically visible copy number variants (CNVs), while UBCAs are gains or losses of euchromatic material in the size of megabasepairs, where according to sheer size of the imbalance a severe phenotype would have to be expected. Still, in cases characterized as having a UBCA, severe phenotypes remain missing, and carrier of an UBCA show no or only minor symptoms [4].

For short arm of chromosome 18 it is known that partial tetrasomy 18p (iso-chromosome 18p syndrome; OMIM **#** 614,290) leads to a severe phenotype, while trisomy of the same region only impairs such carriers comparatively mild [5]. On the other hand there is a 18p- syndrome (OMIM **#** 146,390), which impairs the carriers when the shortened 18p-arm can be clearly identified in GTG-banding. Prenatally, such cases may be recognized due to increased nuchal translucency or holoprosencephaly (HPE) [6, 7]. However, as already highlighted before, the short arm of chromosome 18 is a genomic region with potential to form UBCAs [4, 8–10].

Here we report the characterization of a two-generation family with an in GTG-banding cryptic UBCA in 18p11.32 to 18p11.31 of 4.4. Mb in size. The first hint towards that came from noninvasive prenatal testing (NIPT).

## Case presentation

**Fig. 1:**
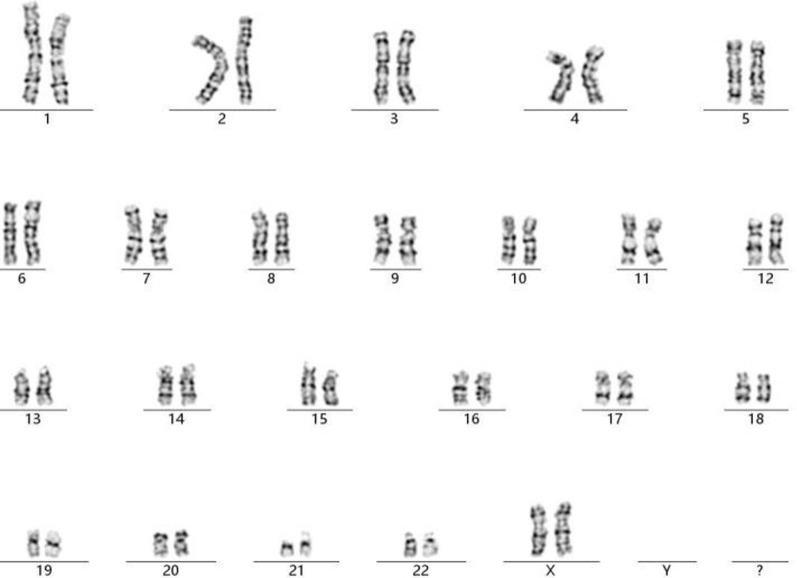
GTG-banding result of the fetus with the cryptic del(18)p11.31

**Fig. 2:**
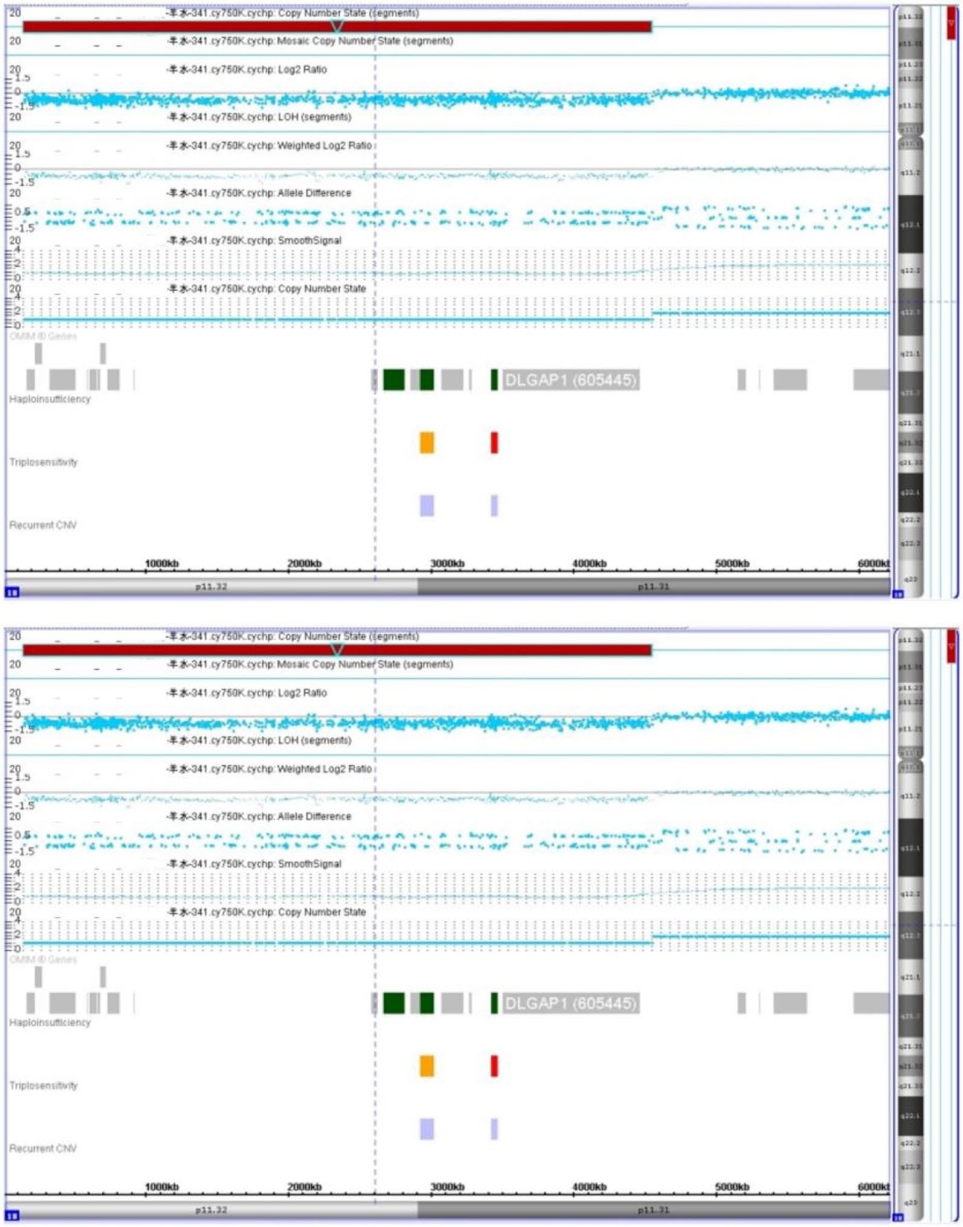
Depiction of the terminal 4.4-Mb deletion revealed by CMA (red marked region); the final result for the fetus was 46,XX.arr[GRCh37] 18p11.32p11.31(136,228_4,538,224) × 1

A 34-year-old, gravida 1, para 0 pregnant woman underwent amniocentesis at 18 weeks of gestation because result of a genome wide NIPT screening gave a hint for a 4.4 Mb microdeletion encompassing 18p11.32 to 18p11.31. Her husband was 35 years old and no family history of birth defects or genetic diseases was reported. The cytogenetic analysis of the cultured amniocytes revealed a normal female karyotype of 46,XX (Fig. 1). Chromosomal microarray analysis (CMA) of DNA derived from uncultured amniocytes was performed using Affymetrix CytoScan 750 K chip, which included 550 k nonpolymorphic markers and 200 k single-nucleotide polymorphism markers. CMA confirmed the presence of the 4.4-Mb chromosomal deletion, which is to be reported according to International System of Cytogenomic Nomenclature 2020 (ISCN 2020) [10] as arr[GRCh37] 18p11.32p11.31(136,228_4,538,224) × 1 (Fig. 2).

Parental karyotypes were done and were 46,XX and 46,XY, respectively. However, in CMA the mother had the same deletion in 18p as the fetus. Ultrasound examination showed no dysmorphisms or intrauterine growth restrictions (IUGRs) in the fetus. After genetic counseling, the parents decided to continue the pregnancy. At 39 weeks of gestation, the mother gave birth vaginally to a female baby. The baby's growth parameters at birth were in the normal ranges. The Apgar scores were 9/9/10. The results of complete physical examination were normal. At 36-month follow-up, the baby was developing normally (intelligence quotient, IQ = 109).

## Discussion

According to the literature [4, 8–10] yet only three cases/ families with a del(18)(p11.3) are reported, which did not show any or minimal clinical signs. Here a third case with clearly characterized size of 4.4 Mb is added to the literature. This highlights the necessity to be careful in hasty conclusions about the potential impact of gains or losses as detected in NIPT or CMA analyses. Without a parental genetic test and best also a GTG-banding the nature and impact of a detected imbalance cannot be interpreted reliably.

Still it is interesting and needs further investigations that the in the reported family deleted region in 18p11.3 encompasses 19 genesas *USP14*, *THOC1*, *COLEC12*, *CETN1*, *CLUL1*, *TYMS*, *ENOSF1*, *YES1*, *ADCYAP1*, *METTL4*, *NDC80*, *SMCHD1*, *EMILIN2*, *LPIN2*, *MYOM1*, *MYL12A-B*, *TGIF1*, and *DLGAP1*. Twelve dose-sensitive genes existed in the short arm of chromosome 18 [12], with *TGIF1* being one of them. Mutations in or absence of *TGIF1* can cause HPE, anencephaly, and pituitary dysplasia. *TGIF1* is expressed in the fetal and adult nervous system, and its deletions have been related to diseases of the central nervous system. The gene regulates neuronal development, patterning, and survival, as well as fetal neuronal axis development in early embryogenesis. *TGIF1* gene mutation and deletion have been associated with autosomal dominant mode of inheritance for HPE [13]. However, *TGIF1* mutations have also been reported in normal individuals and patients with mental retardation or those showing a very mild phenotype, suggesting incomplete penetrance and variable expressivity [14].

## Conclusions

With this report it is highlighted that (sub)chromosomal imbalances like microdeletion in 18p11.3 may show great variability concerning phenotypic consequences. The problem is even worse in prenatal cases, as presented here, as not all phenotypic effects of a genomic imbalance may be prenatally accessible. Overall, cases like the present remind that parental testing is always necessary, also in cases of imbalances being megabasepairs in size, not to miss UBCAs and terminate a potentially healthy offspring.

## Data Availability

Please contact the corresponding author for data requests.

## Abbreviations

CNVs: Copy number variants
CMA: Chromosomal microarray analysis
UBCA: Unbalanced chromosome abnormality
NIPT: Noninvasive prenatal testing
IUGRs: Intrauterine growth restrictions
